# Supplementary material for: Comprehensive cell type decomposition of circulating cell-free DNA with CelFiE
Source: Nat Commun. 2021 May 11;12:2717. doi: 10.1038/s41467-021-22901-x (PMC8113516; doi:10.1038/s41467-021-22901-x)
Supplement: Supplementary file 1 — Supplementary Information [file 41467_2021_22901_MOESM1_ESM.pdf]

Supplemental Note

EM algorithm details for one sample

The full data likelihood is:

$$P(x, z, Y|\alpha, \beta) = P(x|z, \beta)P(z|\alpha)P(Y|\beta) \tag{16}$$

Where the first term is:

$$\log P(x|z, \beta) = \sum_{t,m,c} \log P(x_{mc}|z_{tmc}, \beta_{tm}) \tag{17}$$

$$\equiv \sum_{t,m,c} \log \left[(\beta_{tm})^{z_{tmc}\cdot x_{mc}} (1 - \beta_{tm})^{z_{tmc}\cdot(1-x_{mc})}\right] \tag{18}$$

$$\equiv \sum_{t,m,c} z_{tmc} \left[x_{mc} \log (\beta_{tm}) + (1 - x_{mc}) \log (1 - \beta_{tm})\right] \tag{19}$$

The second term is:

$$\log P(z|\alpha) = \sum_{t,m,c} \log P(z_{tmc}|\alpha) = \sum_{t,m,c} \log (\alpha_t^{z_{tmc}}) = \sum_{t,m,c} z_{tmc} \log \alpha_t \tag{20}$$

The final term is:

$$\log P(Y|\beta) = \sum_{t,m} \left(Y_{tm} \log \beta_{tm} + (D^Y_{tm} - Y_{tm}) \log(1 - \beta_{tm})\right) \tag{21}$$

We calculate the Q function using the conditional distribution for z given some α, β, and the observed reads x:

$$P(z_{tmc} = 1|x_{mc}, \beta, \alpha) \propto P(x_{mc}|z_{tmc} = 1, \beta)P(z_{tmc} = 1|\alpha) \propto \left(\beta_{tm}^{x_{mc}} (1 - \beta_{tm})^{1-x_{mc}}\right) \alpha_t \implies \tag{22}$$

$$P(z_{tmc} = 1|x_{mc}, \beta, \alpha) = \frac{\left(\beta_{tm}^{x_{mc}}(1 - \beta_{tm})^{1-x_{mc}}\right) \alpha_t}{\sum_k \left(\beta_{kt}^{x_{mc}}(1 - \beta_{kt})^{1-x_{mc}}\right) \alpha_k} =: p_{tmc}(\alpha, \beta) \tag{23}$$

The second line follows from the fact that $\sum_t P(z_{tmc} = 1|\cdot) = 1$, as every read must come from some cell type.

The Q-function can only have one of two values depending on the methylation state of x_{mc}:

$$\frac{\beta_{tm}\alpha_t}{\sum_k \beta_{kt}\alpha_k} =: p_{tm1}(\alpha, \beta) = p_{tmc}(\alpha, \beta) \text{ if } x_{mc} = 1 \tag{24}$$

$$\frac{(1 - \beta_{tm})\alpha_t}{\sum_i (1 - \beta_{kt})\alpha_k} =: p_{tm0}(\alpha, \beta) = p_{tmc}(\alpha, \beta) \text{ if } x_{mc} = 0 \tag{25}$$

E step: The Q function is defined at iteration i by:

$$Q_i(\beta, \alpha) := \mathbb{E}_{z|x,\alpha^{(i)},\beta^{(i)}} (\log P(x, z, y|\alpha, \beta)) \tag{26}$$

To evaluate this, we break it into three parts. Let $p^{(i)}_{tm} := p_{tm1}(\alpha^{(i)}, \beta^{(i)})$–this is just the responsibility function defined above evaluated at the parameter estimates from iteration i. Then:

$$\mathbb{E}_{z|x,\alpha^{(i)},\beta^{(i)}} (\log P(x|z, \alpha, \beta)) \equiv \sum_{t,m,c} \mathbb{E}_{z|x,\alpha^{(i)},\beta^{(i)}} (z_{tmc}) \left[x_{mc} \log (\beta_{tm}) + (1 - x_{mc}) \log (1 - \beta_{tm})\right] \tag{27}$$

$$\equiv \sum_{t,m,c} p^{(i)}_{tmc} \left[x_{mc} \log (\beta_{tm}) + (1 - x_{mc}) \log (1 - \beta_{tm})\right] \tag{28}$$

$$\equiv \sum_{t,m,c} \left[p^{(i)}_{tm1} x_{mc} \log (\beta_{tm}) + p^{(i)}_{tm0}(1 - x_{mc}) \log (1 - \beta_{tm})\right] \tag{29}$$

$$\equiv \sum_{t,m} \left[p^{(i)}_{tm1} x_m \log (\beta_{tm}) + p^{(i)}_{tm0}(D^X_m - x_m) \log (1 - \beta_{tm})\right] \tag{30}$$

The second part is,

$$\mathbb{E}_{z|x,\alpha^{(i)},\beta^{(i)}}\left(\log P(z|\alpha)\right) \equiv \sum_{t,m,c} p_{tmc}^{(i)} \log \alpha_t \tag{31}$$

$$\equiv \sum_{t,m} \left(x_m p_{tm1}^{(i)} + (D_m^X - x_m) p_{tm0}^{(i)} \right) \log \alpha_t \tag{32}$$

The third part is simply binomial sampling, since the cell type is known for each reference read:

$$P(Y|\beta) = \sum_{t,m} \left(Y_{tm} \log \beta_{tm} + (D_{tm}^Y - Y_{tm}) \log(1-\beta_{tm}) \right) \tag{33}$$

Finally, adding the three parts together:

$$Q_i(\beta,\alpha) = \sum_{t,m} \left[p_{tm1}^{(i)} x_m \log(\beta_{tm}) + p_{tm0}^{(i)} (D_m^X - x_m) \log(1-\beta_{tm}) \right] \tag{34}$$

$$+ \sum_{t,m} \left(x_m p_{tm1}^{(i)} + (D_m^X - x_m) p_{tm0}^{(i)} \right) \log \alpha_t \tag{35}$$

$$+ \sum_{t,m} \left(Y_{tm} \log \beta_{tm} + (D_{tm}^Y - Y_{tm}) \log(1-\beta_{tm}) \right) \tag{36}$$

$$= \sum_{t,m} \left[\left(Y_{tm} + p_{tm1}^{(i)} x_m \right) \log(\beta_{tm}) + \left(D_{tm}^Y - Y_{tm} + p_{tm0}^{(i)} (D_m^X - x_m) \right) \log(1-\beta_{tm}) \right] \tag{37}$$

$$+ \sum_{t,m} \left(x_m p_{tm1}^{(i)} + (D_m^X - x_m) p_{tm0}^{(i)} \right) \log \alpha_t \tag{38}$$

M step: First, let $S_K \subset \mathbb{R}^K$ be the probability simplex, and recall the basic fact that for any $a \in \mathbb{R}_{++}^K$:

$$\arg\max_{p \in S_K} \sum_k a_k \log p_k = (a_1, \ldots, a_K) \,/\, \sum_{k=1}^K a_k$$

The standard way to show this is using Lagrange multipliers:

$$\mathcal{L} := \sum_k a_k \log p_k + \lambda \left(1 - \sum_k p_k \right)$$

$$\implies \nabla_{p_k} \mathcal{L} = a_k / p_k - \lambda = 0 \implies p_k^* = a_k \lambda^* \ \forall k$$

$$\implies \nabla_\lambda \mathcal{L} = 1 - \sum_j p_j \implies \sum_j p_j^* = 1 \implies \lambda^* = \frac{1}{\sum_j a_j}$$

$$\implies p_k^* = \frac{a_k}{\sum_j a_j} \ \forall k$$

This is the only critical point of the Lagrangian, and must be a maximum since the sum of concave functions (i.e. $a_k \log p_k$) is concave; moreover, it is feasible since $a \in \mathbb{R}_{++}^K$ by assumption.

From these lines of basic calculus, the α update in (10) follows by taking $a_t = \sum_m \left(x_m p_{tm1}^{(i)} + (D_m^X - x_m) p_{tm0}^{(i)} \right)$. Similarly, the β update in (11) follows by taking $a_1 = p_{tm1}^{(i)} x_m + Y_{tm}$ and $a_2 p_{tm0}^{(i)} (D_m^X - x_m) + D_{tm}^Y - Y_{tm}$.

Supplemental Figures

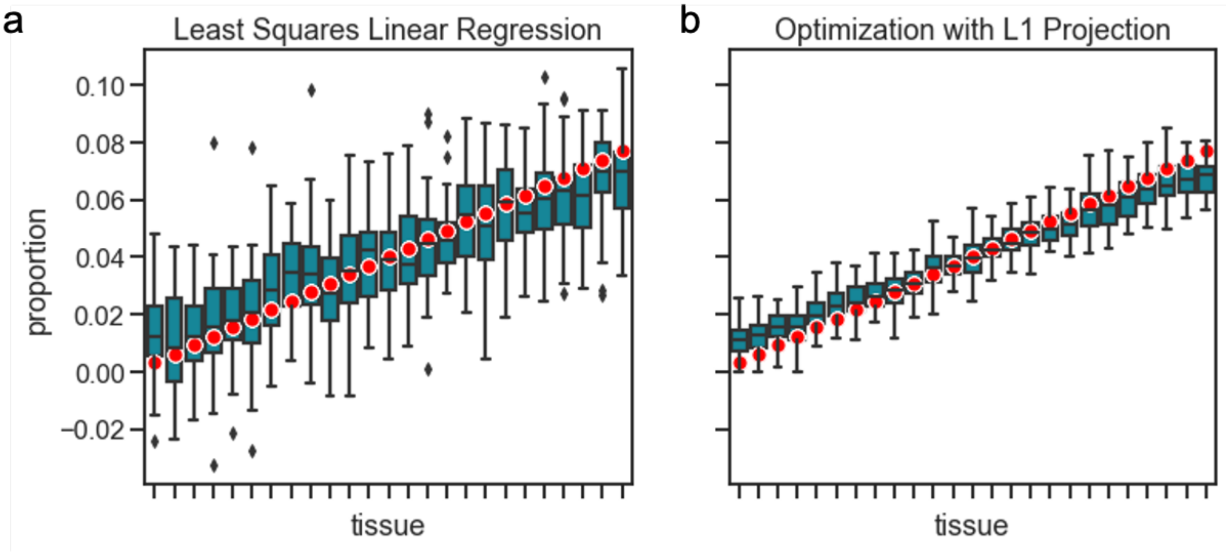

Fig. S1: Decomposition of a single individual's simulated cfDNA mixtures by linear least-squares regression (A) and (B) optimization with an L1 projection. 50 replications were performed, and the estimated mixing proportions were plotted (light blue and dark blue boxes, respectively). True cell type proportions are depicted as red points. The center line of the box indicates the mean, the outer edges of the box indicate the upper and lower quartiles, and the whiskers indicate the maxima and minima of the distribution.

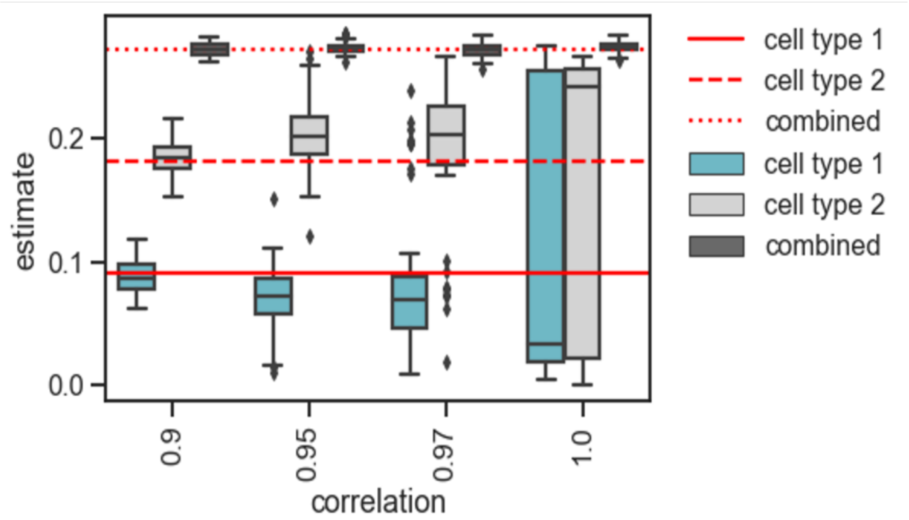

Fig. S2: Decomposition of a single individual's simulated cfDNA mixture containing two correlated cell types. Estimates are shown for each cell type (light blue and grey) along with the sum of the two cell types (dark grey). True cell type proportions are indicated by red lines. The center line of the box indicates the mean, the outer edges of the box indicate the upper and lower quartiles, and the whiskers indicate the maxima and minima of the distribution. Data represents 50 independent simulations.

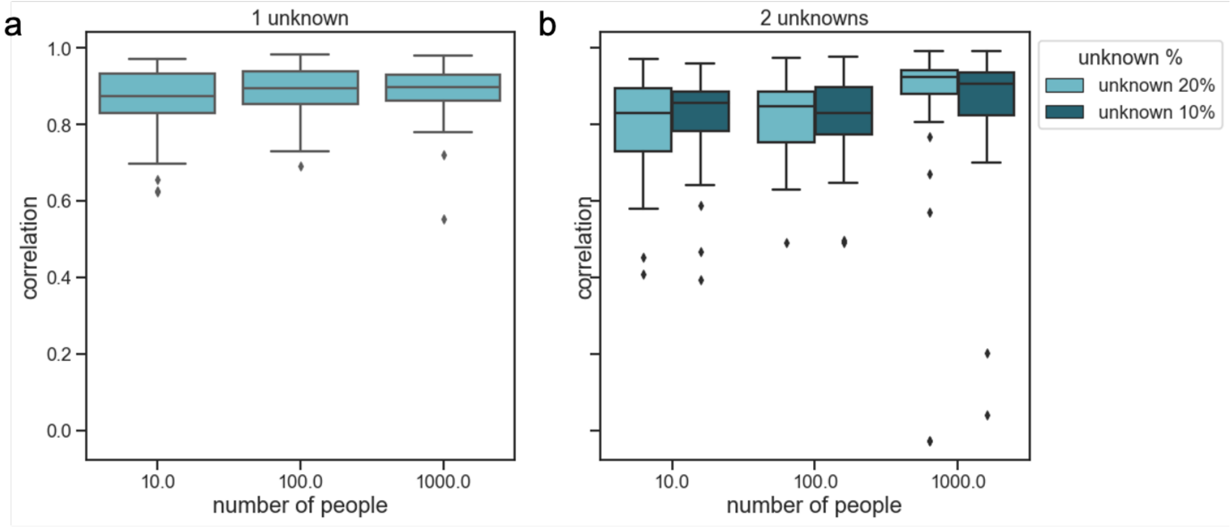

Fig. S3: Correlation between true and CelFiE estimated methylation values. (A) one simulated unknown (light blue boxes) and (B) two simulated unknowns (dark and light blue boxes) for 10, 100, and 1000 people at 10x depth and 1000 CpG sites. Data is shown for 50 independent simulations. In both panels, the center line of the boxplot indicates the mean, the outer edges of the box indicate the upper and lower quartiles, and the whiskers indicate the maxima and minima of the distribution.

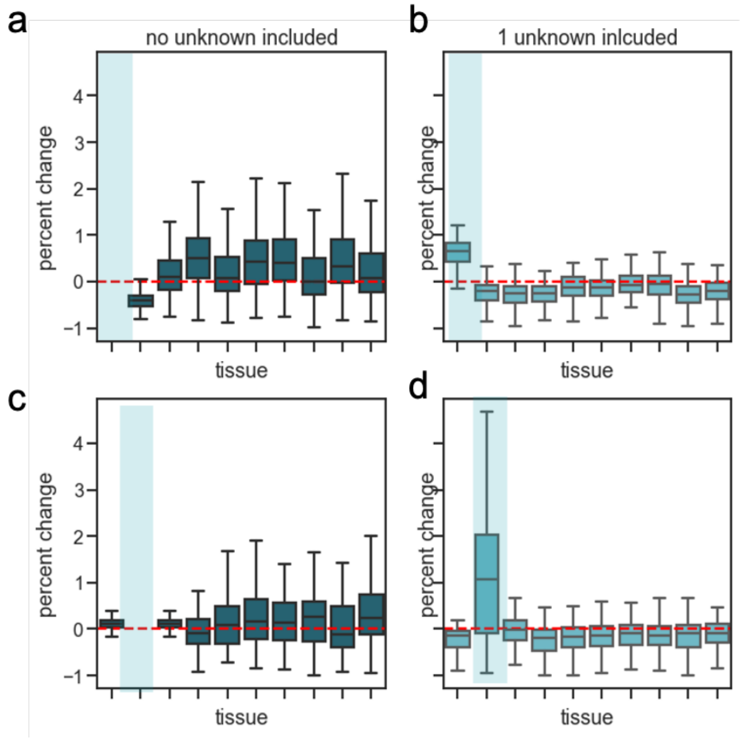

Fig. S4: Percent change of CelFiE estimates from the truth for mixtures with (dark blue boxes) and without an unknown (light blue boxes). 50 independent simulations were performed for 10 individuals at 10x depth. (A) and (B) are mixtures with a missing component of 20% and (C) and (D) are mixtures with a missing component of 10%. Missing cell types are indicated as blue shaded boxes. A percent change of zero, which indicates a correct estimate, is plotted as a red dotted line. A value over the red line is an overestimate relative to the truth, and a value under the red line is an underestimate. The center line of the box indicates the mean, the outer edges of the box indicate the upper and lower quartiles, and the whiskers indicate the maxima and minima of the distribution.

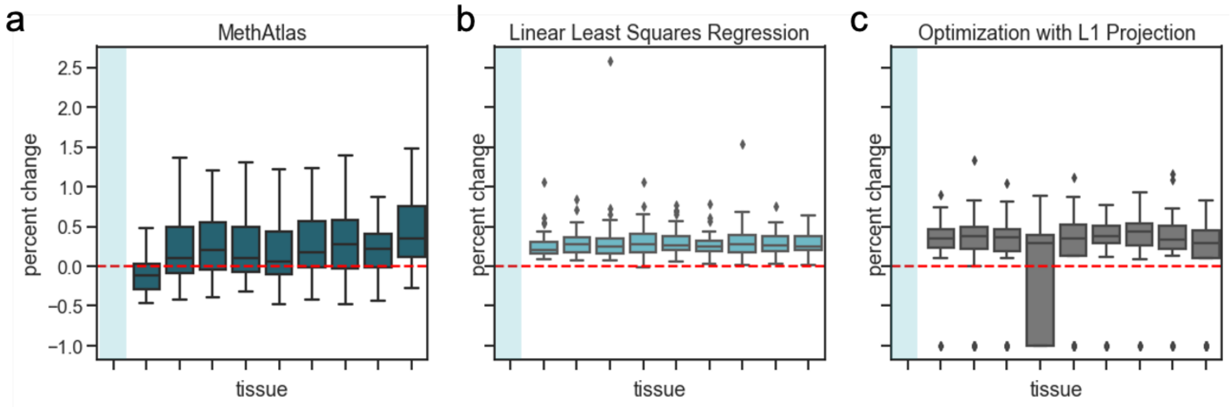

Fig. S5: Performance of (A) MethAtlas (dark blue boxes), (B) least squares regression (light blue boxes), and (C) optimization with an L1 projection (grey boxes) when there is a missing tissue in the reference (indicated by light blue box). Percent change, defined as the difference between the true and estimated proportion, divided by the true proportion, is plotted for of 50 simulation experiments (dark blue, light blue, and grey boxes). The dashed red line indicates a percent change of 0. 50 simulations were performed for simulated cfDNA from 10 individuals at a read depth centered at 10x. In all cases, the center line of the box indicates the mean, the outer edges of the box indicate the upper and lower quartiles, and the whiskers indicate the maxima and minima of the distribution.

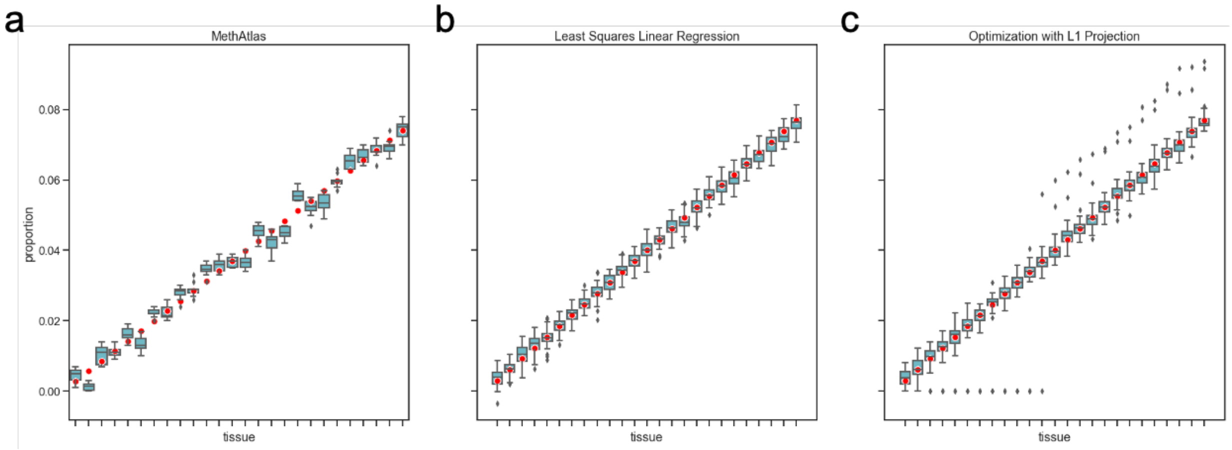

Fig. S6: The performance of (A) MethAtlas (dark blue boxes), (B) least squares linear regression (light blue boxes), and (C) optimization with L1 projection (grey boxes) on simulated data from 1 individual with an average read depth of 100x. 50 simulations were performed. The center line of the boxplot indicates the mean, the outer edges of the box indicate the upper and lower quartiles, and the whiskers indicate the maxima and minima of the distribution.

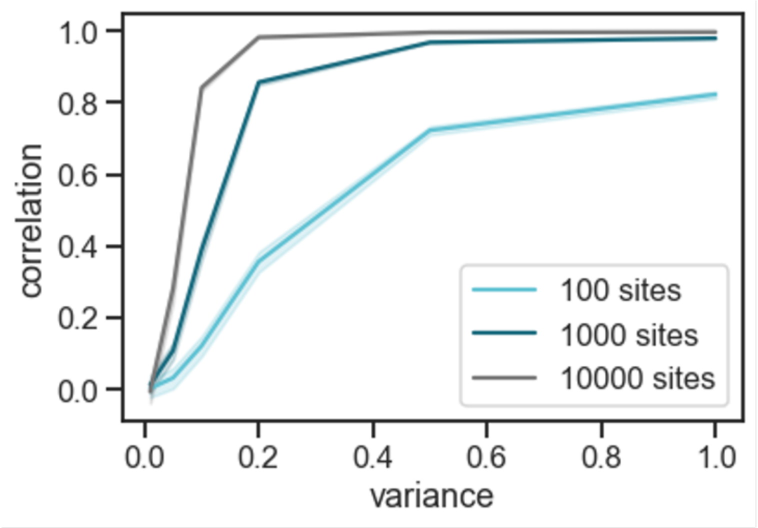

Fig. S7: Correlation between the true cell proportions and CelFiE estimated proportions for a single individual's simulated mixture. The true methylation values are drawn from a normal distribution centered at 0.5, and the variance is allowed to vary between 0 and 1. The higher the variance the more informative a CpG site is for cell type status. Results are shown for 100 sites (light blue line), 1000 sites (dark blue line) and 10000 sites (black line). Data represents 50 independent simulations. The shading around the lines indicates the 95% confidence interval.

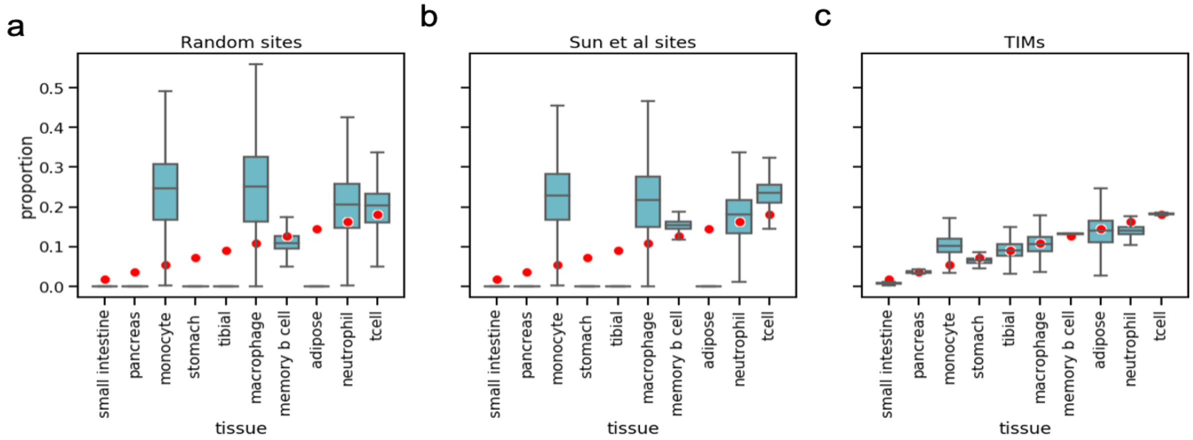

Fig. S8: Performance of CelFie on randomly selected 500 bp regions (A), 500bp regions published in Sun et al [48] (B), and TIMs +/-250bp (C). For one individual's complex simulated cfDNA mixture (red dots) the CelFiE decomposition estimate is plotted (light blue boxes).The center line of the box indicates the mean, the outer edges of the box indicate the upper and lower quartiles, and the whiskers indicate the maxima and minima of the distribution. 50 independent simulations were performed for each set of sites.

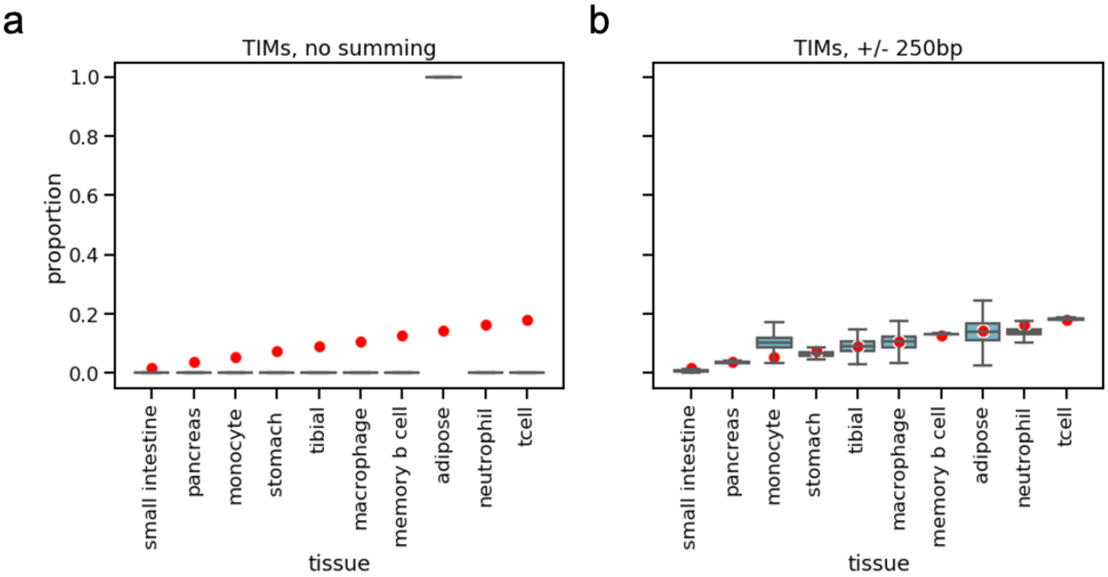

Fig. S9: Performance of CelFie on not summed (A) versus summed sites (B) for a single individual's simulated cfDNA mixture. For a complex mixture of WGBS data (red dots), CelFiE estimates are plotted (light blue). The center line of the box indicates the mean, the outer edges of the box indicate the upper and lower quartiles, and the whiskers indicate the maxima and minima of the distribution. Data represents 50 independent simulations.

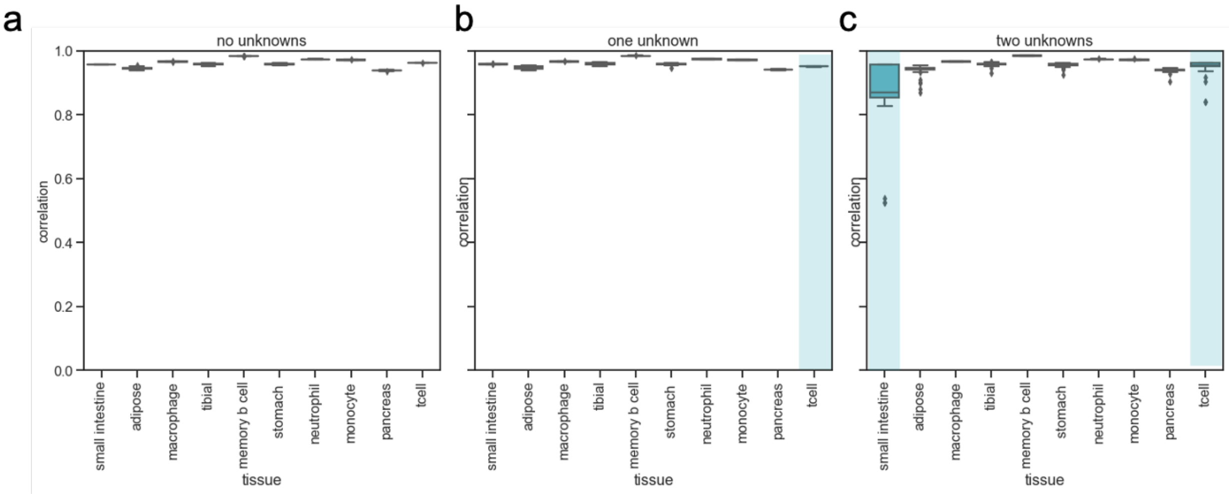

Fig. S10: Correlation between the true and estimated methylation values for n=100 simulated cfDNA mixtures derived from WGBS samples (light blue boxes) when (A) there are no missing cell types, (B) t-cell is missing (indicated by the blue shading) and (C) when both t-cell and small intestine are missing (again indicated by blue shading). The center line of the boxplot indicates the mean, the outer edges of the box indicate the upper and lower quartiles, and the whiskers indicate the maxima and minima of the distribution. Estimates are derived from 50 independent simulations.

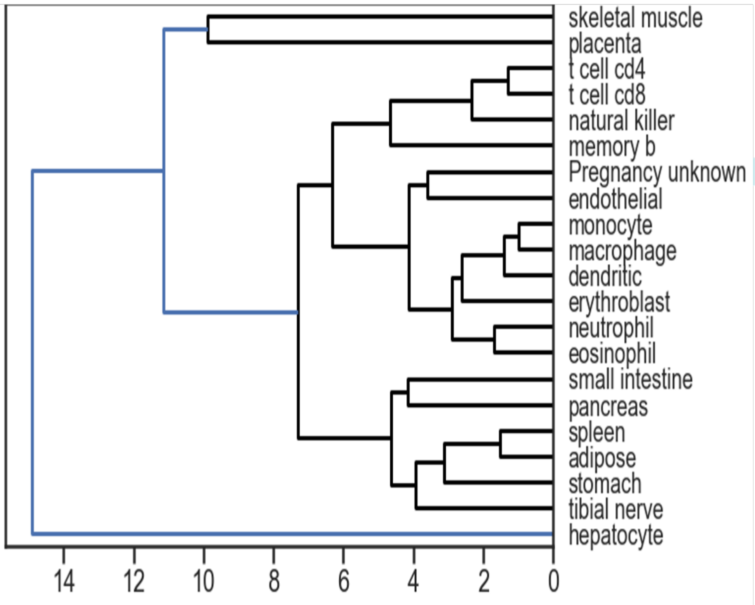

Fig. S11: Hierarchical clustering of the CelFiE unknown component methylation values estimated from n=7 pregnant and n=8 non-pregnant women. The dark blue and black colors indicate clusters detected by the hierarchical clustering algorithm.

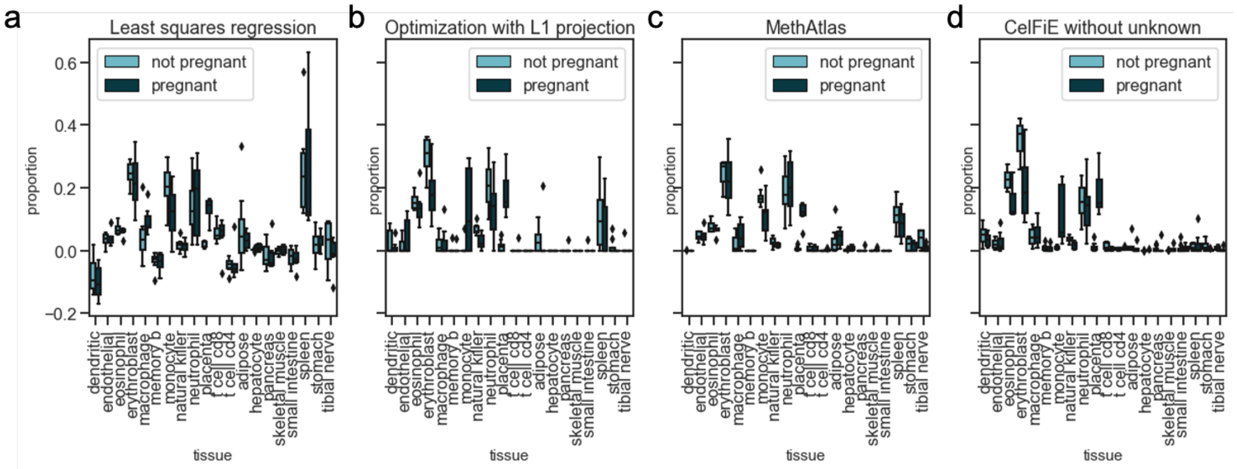

Fig. S12: Decomposition estimates from (A) linear least squares, (B) optimization with L1 projection, (C) MethAtlas, and (D) CelFiE ran without an unknown for n=8 non-pregnant (light blue) and n=7 pregnant women (dark blue). For all four panels, the center line of the box indicates the mean, the outer edges of the box indicate the upper and lower quartiles, and the whiskers indicate the maxima and minima of the distribution.

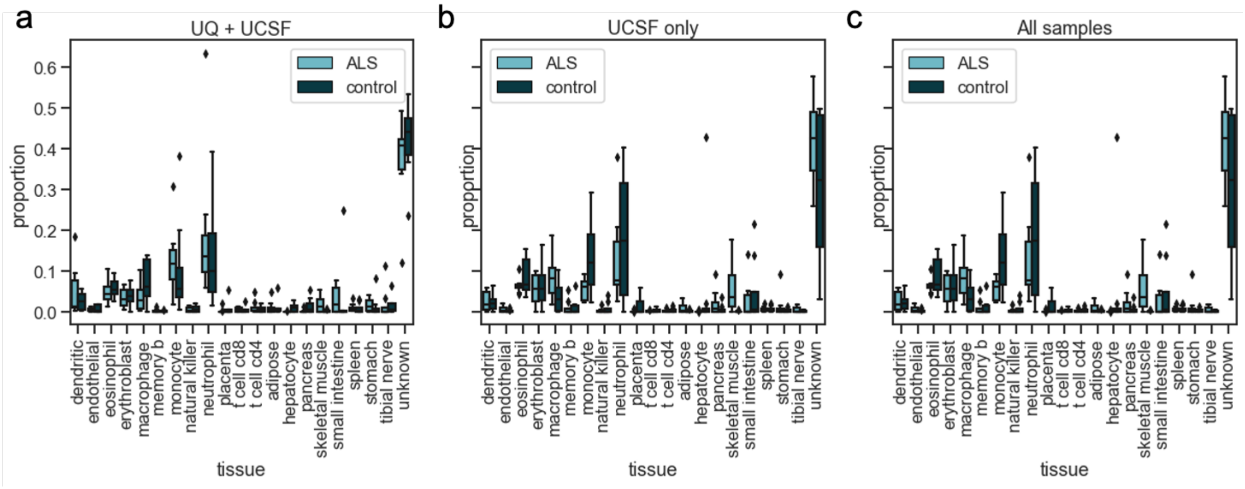

Fig. S13: CelFiE estimates for ALS patients and age-matched controls. (A) UCSF cohort of n=8 cases and n=8 controls (B) Cohort of n=4 cases and n=4 controls from UCSF and n=4 cases and n=4 controls from UQ (total n=8 cases and n=8 controls) (C) all samples from UCSF and UQ cohorts fit jointly (total n=16 cases and n=16 controls. Light blue boxes indicate ALS cases and dark blue boxes indicate controls. In each case, the center line of the box indicates the mean, the outer edges of the box indicate the upper and lower quartiles, and the whiskers indicate the maxima and minima of the distribution.

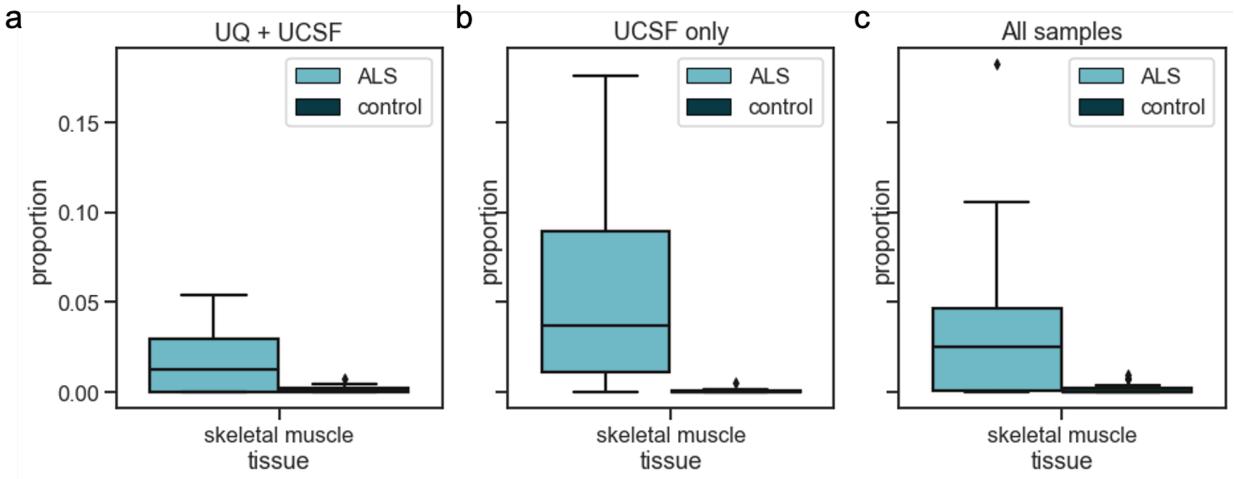

Fig. S14: CelFiE estimates for skeletal muscle. (A) UCSF cohort of n=8 cases and n=8 controls (B) cohort of n=4 cases and n=4 controls from UCSF and n=4 cases and n=4 controls from UQ (n=8 total cases and n=8 total controls) (C) all samples from UCSF and UQ cohorts fit jointly (n=16 total cases and n=16 total controls). Light blue boxes indicate ALS cases and dark blue boxes indicate controls. In each case, the center line of the box indicates the mean, the outer edges of the box indicate the upper and lower quartiles, and the whiskers indicate the maxima and minima of the distribution.

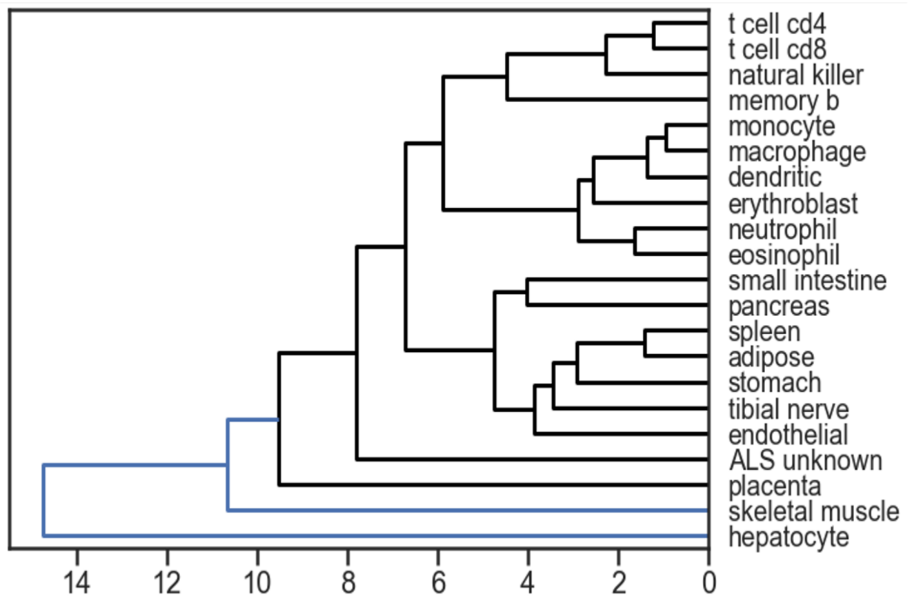

Fig. S15: Hierarchical clustering of the CelFiE unknown component methylation values estimated from n=16 ALS cases and n=16 controls. Blue and black coloring indicates distinct clusters detected by the hierarchical clustering algorithm.

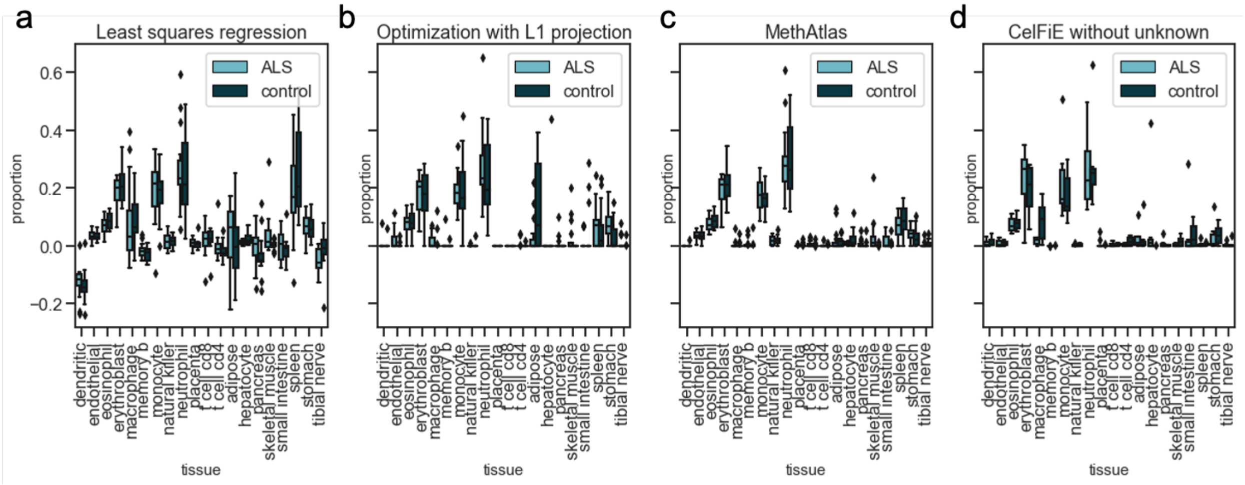

Fig. S16: Decomposition estimates for n=16 ALS patients (light blue) and n=16 controls (dark blue). (A) linear least squares regression, (B) optimization with L1 projection, (C) MethAtlas, and (D) CelFiE ran without an unknown for . For all four panels, the center line of the box indicates the mean, the outer edges of the box indicate the upper and lower quartiles, and the whiskers indicate the maxima and minima of the distribution.

Supplemental Tables

	Linear Least Squares	Optimization with L1 Projection	MethAtlas
**Pregnant**	0.13 ± 0.035	0.19 ± 0.072	0.12 ± 0.033
**Not Pregnant**	0.017 ± 0.0115	0.016 ± 0.018	$5.8 \times 10^{-3} \pm 7.3 \times 10^{-3}$

Table S1: Placenta estimates for n=8 non-pregnant and n=7 pregnant women by linear least squares regression, our projection optimization method, and MethAtlas.

	Linear Least Squares	Optimization with L1 Projection	MethAtlas
**ALS**	0.041 ± 0.075	0.028 ± 0.058	0.038 ± 0.060
**Controls**	$3.1 \times 10^{-3} \pm 0.01$	0.0 ± 0.0	$1.1 \times 10^{-3} \pm 3.4 \times 10^{-3}$

Table S2: Skeletal muscle estimates for n=16 ALS patients and n=16 controls by linear least squares regression, our projection optimization method, and MethAtlas.
