# Supplementary material for: Comprehensive cell type decomposition of circulating cell-free DNA with CelFiE
Source: Nat Commun. 2021 May 11;12:2717. doi: 10.1038/s41467-021-22901-x (PMC8113516; doi:10.1038/s41467-021-22901-x)
Supplement: Supplementary file 3 — Description of Additional Supplementary Files [file 41467_2021_22901_MOESM3_ESM.pdf]

Description of Additional Supplementary Files

File Name: Supplementary Data 1
Description: ENCODE and BLUEPRINT WGBS accession codes and links.
