# Supplementary material for: Comprehensive cell type decomposition of circulating cell-free DNA with CelFiE
Source: Nat Commun. 2021 May 11;12:2717. doi: 10.1038/s41467-021-22901-x (PMC8113516; doi:10.1038/s41467-021-22901-x)
Supplement: Supplementary file 5 — Reporting Summary [file 41467_2021_22901_MOESM5_ESM.pdf]

nature research

Corresponding author(s): Christa Caggiano, Noah Zaitlen

Last updated by author(s): 3/02/2021

Reporting Summary

Nature Research wishes to improve the reproducibility of the work that we publish. This form provides structure for consistency and transparency in reporting. For further information on Nature Research policies, see our Editorial Policies and the Editorial Policy Checklist.

Statistics

For all statistical analyses, confirm that the following items are present in the figure legend, table legend, main text, or Methods section.

n/a	Confirmed	
☐	☒	The exact sample size (*n*) for each experimental group/condition, given as a discrete number and unit of measurement
☐	☒	A statement on whether measurements were taken from distinct samples or whether the same sample was measured repeatedly
☐	☒	The statistical test(s) used AND whether they are one- or two-sided<br>*Only common tests should be described solely by name; describe more complex techniques in the Methods section.*
☐	☒	A description of all covariates tested
☐	☒	A description of any assumptions or corrections, such as tests of normality and adjustment for multiple comparisons
☐	☒	A full description of the statistical parameters including central tendency (e.g. means) or other basic estimates (e.g. regression coefficient) AND variation (e.g. standard deviation) or associated estimates of uncertainty (e.g. confidence intervals)
☐	☒	For null hypothesis testing, the test statistic (e.g. *F*, *t*, *r*) with confidence intervals, effect sizes, degrees of freedom and *P* value noted<br>*Give P values as exact values whenever suitable.*
☒	☐	For Bayesian analysis, information on the choice of priors and Markov chain Monte Carlo settings
☒	☐	For hierarchical and complex designs, identification of the appropriate level for tests and full reporting of outcomes
☐	☒	Estimates of effect sizes (e.g. Cohen's *d*, Pearson's *r*), indicating how they were calculated

Our web collection on statistics for biologists contains articles on many of the points above.

Software and code

Policy information about availability of computer code

Data collection	No software was used for data collection.
Data analysis	WGBS processing used the open source packages Bowtie 2 (v 2.3.5.1), Bismark (v 0.23.0), FastQC (v 0.11.9), TrimGalore (v 0.6.6), Samtools (v 1.7), and MethylDackel (v 0.5.0) . Our linear-least squares regression approach uses SciPy (v 1.5.2). The L1 projection method was implemented as a custom Python script using the SciPy minimize package and the l-bfgs-b algorithm. The projection method code is included on the CelFiE Github. CelFiE uses python 3 and numpy (v 1.19.1), bottleneck (v 1.3.2), and pandas (v 1.2.0) packages. Figures were generated also using python 3 and the matplotlib (v 3.3.1) and seaborn (v 0.10.1) packages.<br><br>A conda environment file is provided in the CelFiE github repository that creates an environment suitable for running CelFiE.<br><br>More details can be found in the CelFiE github repository: https://github.com/christacaggiano/celfie. MethAtlas was accessed from https://github.com/nloyfer/meth_atlas.

For manuscripts utilizing custom algorithms or software that are central to the research but not yet described in published literature, software must be made available to editors and reviewers. We strongly encourage code deposition in a community repository (e.g. GitHub). See the Nature Research guidelines for submitting code & software for further information.

Data

Policy information about availability of data

All manuscripts must include a data availability statement. This statement should provide the following information, where applicable:

- Accession codes, unique identifiers, or web links for publicly available datasets
- A list of figures that have associated raw data
- A description of any restrictions on data availability

ALS cfDNA generated for this study is available on NCBI GEO with the accession #GSE164600 (https://www-ncbi-nlm-nih-gov.ezproxy.u-pec.fr/geo/query/acc.cgi?acc=GSE164600). Tissue and cell-type WGBS data was obtained from the ENCODE Project (https://www.encodeproject.org/search/?type=Experiment&status=released&perturbed=false) and BLUEPRINT Epigenome Project (http://dcc.blueprint-epigenome.eu/#/files). Pregnancy cfDNA from Jensen et al was obtained from dbGaP accession #phs000846 (https://www.ncbi.nlm.nih.gov/projects/gap/cgi-bin/study.cgi?study_id=phs000846.v1.p1).

Field-specific reporting

Please select the one below that is the best fit for your research. If you are not sure, read the appropriate sections before making your selection.

- [x] Life sciences
- [ ] Behavioural & social sciences
- [ ] Ecological, evolutionary & environmental sciences

For a reference copy of the document with all sections, see nature.com/documents/nr-reporting-summary-flat.pdf

Life sciences study design

All studies must disclose on these points even when the disclosure is negative.

Sample size	For the application to existing data sets, we used the largest study available (see Jensen et al 2017). For the ALS application, we exceed the previous largest study (see Mendioroz et al 2018) Also, see main text figure 4 for an examination of deconvolution power as a function of sample size.
Data exclusions	No samples were excluded.
Replication	We replicated findings in our UCSF cohort with a independent cohort from UQ. More details can be found in the main text.
Randomization	The purpose of the application to real data sets was to determine if there are differences between populations. Randomization is not required to perform this analysis.
Blinding	No blinding was used because this was an exploratory study that intended to identify differences between populations.

Reporting for specific materials, systems and methods

We require information from authors about some types of materials, experimental systems and methods used in many studies. Here, indicate whether each material, system or method listed is relevant to your study. If you are not sure if a list item applies to your research, read the appropriate section before selecting a response.

Materials & experimental systems

n/a	Involved in the study	
x		Antibodies
x		Eukaryotic cell lines
x		Palaeontology and archaeology
x		Animals and other organisms
	x	Human research participants
x		Clinical data
x		Dual use research of concern

Methods

n/a	Involved in the study	
x		ChIP-seq
x		Flow cytometry
x		MRI-based neuroimaging

Human research participants

Policy information about studies involving human research participants

Population characteristics	ALS patients and non-related age matched controls were recruited from the UCSF ALS Center and the University of Queensland ALS Clinic. Patients were identified as ALS cases by expert neurologists. The mean age of subjects was 60.7 +/- 14.7 years, and the subjects were 50% female.
Recruitment	Subjects were recruited and evaluated by expert physicians. Study participation was voluntary. Blood extraction for the cfDNA collection was part of a patient's routine clinic visit. We do not believe there was possibility for self-selection bias as cases spanned the disease spectrum and controls were their partners.
Ethics oversight	Written informed consent was obtained from all study participants. Study was approved by the UCSF (IRB 10-05027) and UQ institutional review boards (IRB 2018002470).

Note that full information on the approval of the study protocol must also be provided in the manuscript.
